# Fractal-Based Architectures with Skip Connections and Attention Mechanism for Improved Segmentation of MS Lesions in Cervical Spinal Cord

**DOI:** 10.3390/diagnostics15081041

**Published:** 2025-04-19

**Authors:** Rukiye Polattimur, Mehmet Süleyman Yıldırım, Emre Dandıl

**Affiliations:** 1 Department of Electronics and Computer Engineering, Institute of Graduate, Bilecik Seyh Edebali University, 11230 Bilecik, Türkiye; rukiye.polattimur@bilecik.edu.tr; 2 Department of Computer Technology, Söğüt Vocational School, Bilecik Şeyh Edebali University, Sögüt, 11600 Bilecik, Türkiye; mehmets.yildirim@bilecik.edu.tr; 3 Department of Computer Engineering, Faculty of Engineering, Bilecik Seyh Edebali University, 11230 Bilecik, Türkiye

**Keywords:** cervical spinal cord, multiple sclerosis, automatic segmentation, U-Net, FractalSpiNet, Con-FractalU-Net, Att-FractalSpiNet, MRI

## Abstract

**Background/Objectives**: Multiple sclerosis (MS) is an autoimmune disease that damages the myelin sheath of the central nervous system, which includes the brain and spinal cord. Although MS lesions in the brain are more frequently investigated, MS lesions in the cervical spinal cord (CSC) can be much more specific for the diagnosis of the disease. Furthermore, as lesion burden in the CSC is directly related to disease progression, the presence of lesions in the CSC may help to differentiate MS from other neurological diseases. **Methods**: In this study, two novel deep learning models based on fractal architectures are proposed for the automatic detection and segmentation of MS lesions in the CSC by improving the convolutional and connection structures used in the layers of the U-Net architecture. In our previous study, we introduced the FractalSpiNet architecture by incorporating fractal convolutional block structures into the U-Net framework to develop a deeper network for segmenting MS lesions in the CPC. In this study, to improve the detection of smaller structures and finer details in the images, an attention mechanism is integrated into the FractalSpiNet architecture, resulting in the Att-FractalSpiNet model. In addition, in the second hybrid model, a fractal convolutional block is incorporated into the skip connection structure of the U-Net architecture, resulting in the development of the Con-FractalU-Net model. **Results:** Experimental studies were conducted using U-Net, FractalSpiNet, Con-FractalU-Net, and Att-FractalSpiNet architectures to detect the CSC region and the MS lesions within its boundaries. In segmenting the CSC region, the proposed Con-FractalU-Net architecture achieved the highest Dice Similarity Coefficient (DSC) score of 98.89%. Similarly, in detecting MS lesions within the CSC region, the Con-FractalU-Net model again achieved the best performance with a DSC score of 91.48%. **Conclusions**: For segmentation of the CSC region and detection of MS lesions, the proposed fractal-based Con-FractalU-Net and Att-FractalSpiNet architectures achieved higher scores than the baseline U-Net architecture, particularly in segmenting small and complex structures.

## 1. Introduction

Segmentation is crucial for visualization and computation in many medical image workflows [1]. For various clinical applications, such as diagnosis, treatment planning, and surgery, medical image segmentation is important. Detection and accurate segmentation of lesions, tumors, and other small anatomical structures are essential for monitoring disease processes and evaluating effective treatment methods [2]. The semantic definition and segmentation of each pixel in medical images is widely used as a decision support system developed for clinical diagnosis, treatment, and pathological evaluation [3]. In particular, the evaluation of diseases such as multiple sclerosis (MS), which directly affects the nervous system and affects the daily life of the person, is one of the most important tools for clinical decision makers [4]. While clinical approaches presented as manual segmentation with expert support can often be quite challenging in terms of time and cost, it is possible to minimize the difficulty of the process by using an automated, reliable, and reproducible decision support system [5,6].

MS is a chronic autoimmune disease of the central nervous system (CNS) that results in demyelination and neurodegeneration [7,8]. The disease manifests primarily through lesions in the brain and spinal cord, with the cervical spinal cord (CSC) playing a critical role in disease progression and disability assessment. MS lesions in the CSC are strongly correlated with motor and sensory impairment, making their accurate detection and segmentation essential for both clinical diagnosis and patient monitoring [9]. MS lesions in the CSC provide important data for predicting disease progression and formulating patient-specific treatment plans. For example, detection of MS lesions in the CSC is important for processes such as predicting response to immunomodulatory therapy, selecting disease-modifying therapy based on lesion burden, and guiding physical therapy and rehabilitation processes.

CSC lesions are directly associated with motor dysfunction, sensory impairment, and disease progression in MS patients [10]. Automated and accurate lesion segmentation can improve early diagnosis, as identification of lesions in the spinal cord can help neurologists confirm the diagnosis of MS. In addition, tracking lesion progression over time allows clinicians to assess disease activity and make timely adjustments to treatment strategies. Furthermore, automated methods minimize inter- and intra-observer variability, ensuring more consistent and objective lesion assessment. Accurate segmentation of MS lesions in the CSC has significant clinical importance in magnetic resonance imaging (MRI). MRI is the gold standard for detection and analysis of MS lesions [11]. The McDonald criteria have provided a set of grading standards for the diagnosis and management of MS disease and have highlighted the importance of MRI, particularly axial T2-weighted (T2-w) scans [12,13]. It is possible to achieve high levels of accuracy in the diagnosis of MS lesions from spinal cord MR images using automated systems developed to support clinical applications [14]. However, the fact that the region of interest (RoI) is located in regions where precision and high quality are required and has small and volumetrically different tissues can create different situations during MR imaging and may have a negative impact on the quality of the data [15]. This situation requires more meticulous and careful data acquisition and dataset generation processes.

There are many computer-aided tools that are used as decision support systems to improve the process of early detection and diagnosis in the clinic [16]. In addition to saving time and labor, these systems can be used as a learning tool for non-specialist or specialist physicians and can be used as a tool to assist in making the correct diagnosis. By using an automated system with deep learning tools, objective and consistent results can be achieved by minimizing human error and enabling clinical practitioners to make decisions with high accuracy. Thus, a more reliable diagnostic protocol and treatment protocol can be established by reducing false rates and ensuring accurate assessment of disease progression.

Automated segmentation approaches based on deep learning have shown promising results in improving accuracy and efficiency. On the other hand, differences in dataset size and quality, variations in model architectures and hyperparameters, inconsistent training and evaluation methods, and a lack of standardized benchmark datasets are major sources of variability in deep learning models for medical image segmentation [17]. In addition, challenges remain due to the small lesion size, low contrast variation, and structural complexity of the spinal cord. Deep learning-based models have been used to segment the spinal cord, particularly in spinal MR images, to identify MS lesions in this region and to perform long-term follow-up analyses. Many previous studies have proposed approaches for the detection and segmentation of spinal cord cross-sectional area (CSA), cerebrospinal fluid (CSF), white matter (WM), grey matter (GM), MS, and other lesion derivatives in the CNS [18,19,20,21]. On the other hand, studies have also been proposed to detect spinal cord regions and textural abnormalities such as lesions and tumors within these boundaries using deep learning-based convolutional networks [21,22,23,24,25,26]. In addition, some studies have been presented for segmentation of the spinal cord, spinal cord GM and WM, and spinal canal using convolutional recurrent neural networks (RNN), ResNet50, and attention mechanism-based deep learning architectures [25,27,28].

In the work proposed by Gros et al. [22], automatic segmentation of spinal cord atrophy and lesions in MS patients was performed. The proposed automatic segmentation approach is based on a two-stage CNN sequence. The first CNN detects the spinal cord center line using 2D extended convolutions, while the second CNN segments the spinal cord and lesions using 3D convolutions. In the study, although a high score was obtained for spinal cord segmentation, the score obtained for MS lesion segmentation was relatively low. In another study, McCoy et al. [23] presented a 2D CNN-based approach for automatic segmentation of spinal cord and contusion injuries from MR images. The developed model was compared with existing best methods. The proposed model showed better performance compared to manual segmentation. However, the use of a small dataset in the study is considered a limitation. Merali et al. [25] developed a deep learning-based model for detection of CSC compression in MRI images. The performance of the proposed CNN model was evaluated after the images were labeled for the presence of spinal cord compression by two expert physicians. The fact that the dataset is limited to a specific group of patients does not give an idea of how the performance of the model may be affected in larger datasets. Horváth et al. [27] proposed a novel multidimensional RNN architecture to automate spinal cord GM and WM segmentation. They presented an approach that enhances texture contrast by obtaining eight different inverse recovery (IR) images of the same anatomical slice. They also compared the results of automated segmentation with manual segmentation but did not report inter-observer agreement rates for manual segmentation. In another study, Perone et al. [21] developed a deep learning-based method for automatic segmentation of spinal cord GM tissue using dilated convolutions. The developed model was compared with six different state-of-the-art methods in GM segmentation tasks, and performance evaluations were performed. However, the study did not evaluate the performance of the model in a larger patient population with different demographic characteristics. Porisky et al. [29] presented a novel method for grey matter segmentation from spinal cord MRI images using 3D convolutional encoder networks and short-cut connections. Although the proposed architecture looks similar to a U-Net structure with encoder, decoder, and shortcut connection, a deconvolution process is used instead of an upsampling process in the decoder part. Naga Karthik et al. [30] developed an open-source 2D and 3D CNN-based tool for automatic segmentation of spinal cord lesions in MS patients from axial T2-w MRI images. The developed tool was evaluated on data obtained from different centers and achieved high accuracy rates in lesion segmentation.

Automatic segmentation of medical images has gained much more momentum, especially with the advent of the U-Net architecture [31]. The U-Net architecture, which provides an end-to-end pixel-based solution, can achieve very successful results even on datasets with a small target area. Although there are few studies on the automatic segmentation of the spinal cord region, spinal cord tumors, and lesions, there are studies using the U-Net architecture, which is developed using a convolutional structure and provides stable and successful results in many aspects, especially in the field of medical imaging. Zhang et al. [26] automated spinal cord segmentation from 2D cervical axial MRI slices. The proposed approach includes a level set-based active contour method by pre-processing MRI images with a U-Net architecture. The number of patients in the study is small, and the performance of the results obtained has not been evaluated with larger and more diverse datasets. In another study, Askari-Hemmat et al. [32] performed grey matter segmentation of the spinal cord using a U-Net architecture based on a fixed-point quantization method. In the study, the quantization process caused a small decrease in the accuracy of the model. Fei et al. [33] achieved automatic segmentation of the internal structures of the CSC using a U-Net model based on pre-trained VGG16 and ResNet50 backbones. In their study, too many RoI’s were identified, resulting in poor performance. Alsenan et al. [34] proposed MobileU-NetV3, a lightweight deep learning model that combines MobileNetV3 and U-Net architectures for spinal cord GM segmentation. The proposed model was evaluated on a specific dataset. Zhang et al. [35] developed the SeUneter architecture for segmentation of cervical MRI spinal structures by deepening the U-Net architecture and adding a channel attention module to the double convolutional layers during feature extraction. The contribution of the channel attention module to the segmentation performance is not analyzed in detail. Bueno et al. [36] proposed an optimized residual attention-aware U-Net architecture for automatic spinal cord segmentation from cervical spine MR images of MS patients. The automatic segmentation model showed some success compared to manual segmentation. In our previous study [37], a novel deep learning architecture called FractalSpiNet was proposed for automatic segmentation of spinal cord and MS lesions in CSC MR images. FractalSpiNet is an architecture based on the U-Net structure and integrates fractal networks for improved feature extraction in MRI scans. The proposed FractalSpiNet architecture has shown better performance in the automatic segmentation task compared to state-of-the-art methods.

In general, work on spinal cord segmentation is limited compared to other medical image segmentation tasks. Several deep learning approaches have been proposed for MS lesion segmentation in the spinal cord. Traditional CNNs have demonstrated success in spinal cord segmentation, but their performance is often limited by a lack of global contextual understanding and difficulties in capturing long-range dependencies. Traditional U-Nets and their variants have been used due to their encoder-decoder architecture and skip connections, which preserve spatial detail. However, these methods often struggle to distinguish small lesions from surrounding tissue due to limited contextual awareness. On the other hand, many studies suffer from the use of small datasets, which can lead to overfitting and limit the generalizability of the model to unseen data. In addition, some studies lack diversity in the data and focus on specific patient populations. Furthermore, studies comparing the proposed methods with existing methods generally show similar performance, suggesting the need for significant improvements in segmentation accuracy, especially for MS lesion detection.

To address these limitations, this study proposes a novel deep learning framework based on fractal architectures with skip connections and an attention mechanism for improved segmentation of MS lesions in the CSC. The proposed fractal-based approach builds on the strengths of existing models while addressing their limitations. Unlike standard CNN-based methods, the fractal architecture enables a hierarchical multi-scale feature extraction, which improves robustness to lesion size variations. Fractal architectures, inspired by self-repeating hierarchical patterns, enable the extraction of multi-scale features, enhancing the network’s ability to capture complex spatial structures. Skip connections facilitate the flow of information across different layers, preserving fine-grained spatial detail and improving gradient propagation. Attention mechanisms are also incorporated to improve the model’s focus on relevant lesion regions, reducing false positives and improving segmentation precision. By leveraging fractal architectures, skip connections, and attention mechanisms, the proposed models aim to provide a robust and efficient approach for automated segmentation of MS lesions in the CSC. This advancement has the potential to assist clinicians in early diagnosis, disease progression monitoring, and treatment planning, ultimately improving patient outcomes. The contributions of this study can be summarized as follows:

1. This study presents two deep learning architectures, Con-FractalU-Net and Att-FractalSpiNet, that utilize fractal convolutional blocks. The use of fractal designs allows the models to explore multiple receptive fields and path depths in parallel, enhancing the network’s ability to learn complex spatial hierarchies. This is particularly valuable in spinal cord imaging, where MS lesions vary widely in size, shape, and intensity.
2. By incorporating U-Net-type skip connections into the fractal architecture, the proposed models maintain fine-grained spatial information across encoding and decoding paths. These connections help mitigate the vanishing gradient problem, especially in deeper networks, and contribute to more accurate lesion delineation by preserving high resolution anatomical details.
3. Att-FractalSpiNet uses attention modules to focus the network on lesion-relevant regions while suppressing less informative background noise. This selective attention strategy enhances the network’s ability to distinguish MS lesions from complex and noisy spinal cord structures, improving both the sensitivity and specificity of the segmentation process.
4. The proposed models were extensively evaluated on a cervical spinal cord MRI dataset, and performance was evaluated using standard metrics. The results show that Con-FractalU-Net and Att-FractalSpiNet outperform conventional architectures such as U-Net and previous fractal-based methods, achieving higher levels of performance, especially in the segmentation of small and irregularly shaped lesions.

The rest of this paper is structured as follows: Section 2 provides a detailed description of the dataset used in this study, along with the preprocessing steps applied to improve data quality and model performance. In addition, this section provides a comprehensive explanation of the proposed Con-FractalU-Net and Att-FractalSpiNet architectures, detailing all their components and innovations. Section 3 focuses on the experimental analysis, where the quantitative metrics obtained from the segmentation and lesion detection tasks are thoroughly evaluated. Furthermore, visualizations of the model outputs are provided to facilitate an in-depth analysis of the segmentation performance. Finally, Section 4 summarizes the experimental findings, elaborates on the study’s conclusions, and discusses the clinical implications of the proposed models. In addition, this section outlines potential future research directions, highlighting areas for further improvements and applications of fractal-based architectures in medical image segmentation.

## 2. Materials and Methods

**Figure 1:**
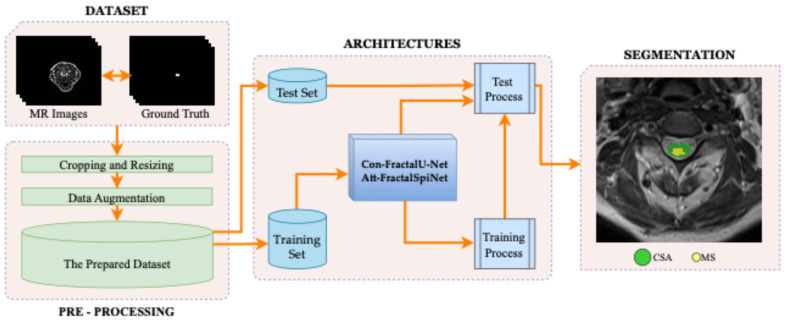
The complete workflow of the proposed segmentation framework for detecting MS lesions in the cervical spinal cord (CSC) using fractal-based deep learning architectures such as skip connections-based Con-FractalU-Net and attention mechanism-based Att-FractalSpiNet.

This study presents fractal-based skip connections-based Con-FractalU-Net and attention mechanism-based Att-FractalSpiNet deep learning approaches for more accurate and successful segmentation of MS lesions in the CSC. In the proposed architectures, a methodology based on fractal convolution-based synthesis U-Net architectures was developed using a CSC dataset consisting of T2-w MR slices. In the study, model training for automatic detection of the CSC region and MS lesions in this region is performed, and the results obtained are compared. The block diagram showing the general methodology of the study is shown in Figure 1. In the first step, the spinal cord MR dataset containing images of the CSC is divided into two subsets as training and test. This dataset is enriched with segmentation annotations, including MRI-based CSC images and MS lesions. Then, since the raw MRI images are not suitable to work directly with deep learning models, some pre-processing techniques were applied. In the second step, since the developed architectures are based on fractal-based U-Net models, the results of our previously proposed FractalSpiNet and basic U-Net architectures trained with specified hyperparameters are compared with the segmentation performance results obtained for the Con-FractalU-Net and Att-FractalSpiNet architectures proposed in this study using key metrics.

### 2.1. Dataset

The cervical part of the human spinal cord is located between the intervertebral discs between the neck and the coccyx. In the clinical setting, it is possible to obtain individual MRI images of the CSC, either regionally or for the entire CSC if needed. As MS lesions often occur in the CSC region, MR scans of the cervical region are performed according to clinical procedures for the detection of MS lesions [38]. The dataset used in the study [39] is a publicly available dataset of T2-w CSC MRI images, performed retrospectively, containing samples of healthy and MS patients, labeled with manual segmentation masks, and used in our previous study [37]. The images in the dataset were generated from 2D MR slices of the CSC region of 87 MS patients, 68 females and 19 males, scanned axially in the turbo spin echo sequence and T2-w modality. T2-w images are critical for segmentation of the spinal cord and MS lesions, as they facilitate the separation of WM and GM and the detection of pathological structures.

**Figure 2:**
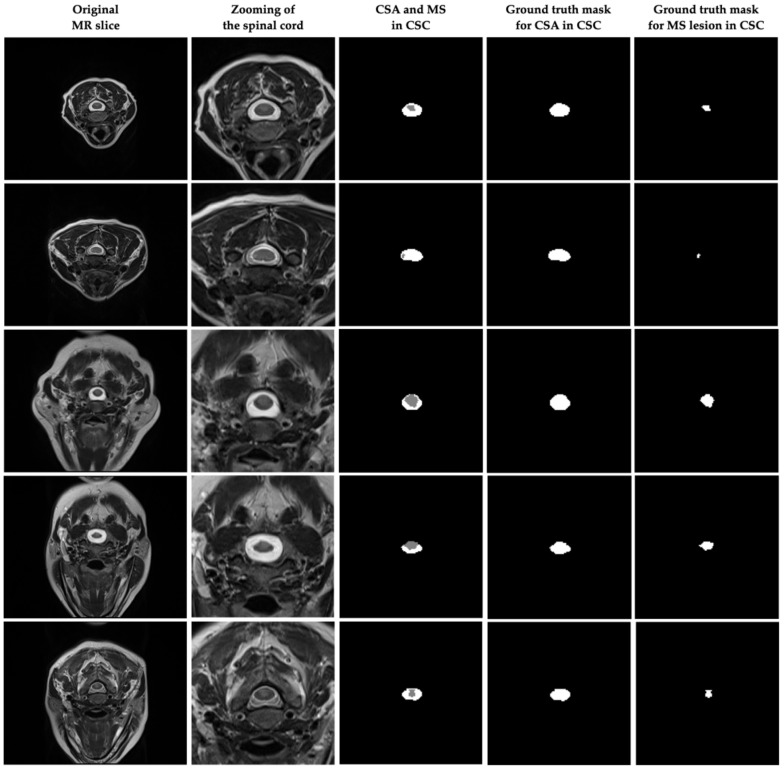
Some of the original MR images in the dataset used in the study and ground truth masks of CSA and MS lesions in CSC.

The images in the scans obtained from the SIEMENS Spectra Magnetom 3T device as DICOM in the dataset were 320 × 250 pixels, and the slice thickness in the scans was 4 mm. For the cross-sectional area (CSA) and MS lesions in the images of the MR scans forming the dataset, ground truth masks were manually determined by two different radiologists using ITK-SNAP 4.0 software [40]. This resulted in a total of 231 suitable slices for CSA and MS in the axial plane. Preprocessing steps and data augmentation techniques were used to train the model, and performance analysis was performed by dividing the data into training, validation, and test sets. Figure 2 shows some of the original MR images in the dataset and the ground truth masks of the CSA and MS lesions in CSC. In addition, it was approved by the decision of the Clinical Research Ethics Committee of Akdeniz University Faculty of Medicine dated 15 September 2021 and numbered KAEK-644 that there are no ethical objections to conducting this study and creating the dataset.

### 2.2. Proposed Methodology

Many deep learning models have been developed for medical imaging. Deep learning has revolutionized medical image analysis, especially in segmentation tasks where accurate delineation of anatomical structures and pathological regions is crucial. Among deep learning models, with the development of the convolutional U-Net architecture in medical segmentation studies, it has become a standard architecture for biomedical image segmentation due to its encoder-decoder structure and skip connections, which allow precise localization by preserving spatial information [5,41]. Due to its successful segmentation capability and easy model synthesis, various U-Net architectures have been developed to solve many problems [42]. However, conventional U-Net models can struggle with complex structures such as the CSC and MS lesions due to variations in shape, intensity, and contrast. To address these challenges, hybrid U-Net architectures have gained significance by integrating advanced mechanisms such as fractal designs, attention modules, and skip connections. These enhancements improve feature representation, enable better learning of fine-grained details, and increase robustness against variations in medical images. In this study, in addition to our previously proposed FractalSpiNet architecture [37], we propose two new models, such as Con-FractalU-Net and Att-FractalSpiNet, specifically designed for the automatic segmentation of the CSC and MS lesions in MRI scans.

#### 2.2.1. U-Net

**Figure 3:**
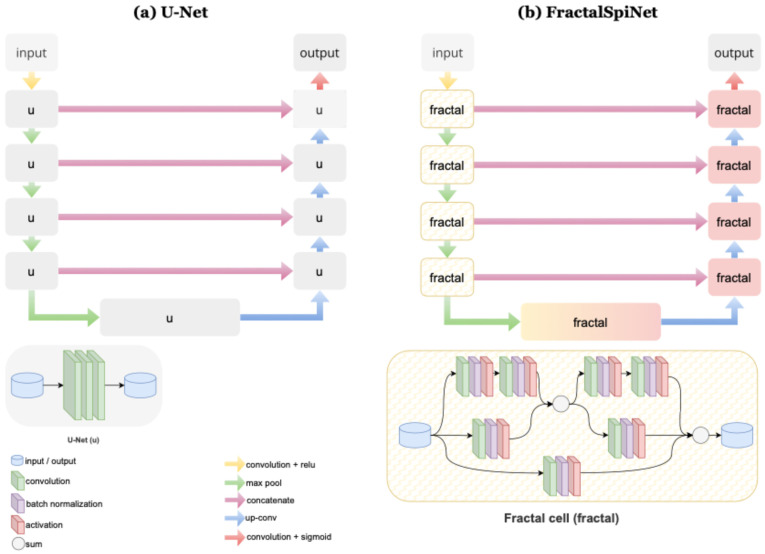
Details of the architectures used in the study and the components that comprise these architectures. (**a**) U-Net, (**b**) FractalSpiNet.

U-Net is a CNN-based deep learning architecture and a successful method developed specifically for image segmentation [41]. In the U-Net architecture, important features in the input image are extracted using convolutional layers, and then the class/labeling of each pixel is predicted using these features. The U-Net deep learning architecture consists of two parts, an encoder and a decoder, connected by a bottleneck [43]. In the encoder stage, the image is reduced to more abstract and low-dimensional features using convolutional layers, while in the decoder stage, the low-dimensional features obtained by inverse convolution are converted back to high resolution. In the decoder stage, the upward inverse convolution process performed on the contraction path in the contraction path increases the segmentation success by gradually increasing the sensitive feature data. In addition, thanks to the skip connections in the structure of the architecture, low-level features obtained from the encoder are directly transferred to the decoder to increase the segmentation accuracy [44]. U-Net enables the effective use of convolutional networks, especially in classification and segmentation. Although U-Net is based entirely on a convolutional network, it differs in that it has a U-shaped symmetric architecture, as shown in the typical U-Net architecture in Figure 3a, and uses skip connections between the encoder and decoder subnetworks to combine low-level and high-level features to preserve more refined image details.

The U-Net architecture performs the extraction of the specific region to be segmented on the images and provides superior segmentation performance with less data compared to other deep learning models with advanced feature selection. Although the basic U-Net model has a very strong performance, it still needs to be improved for challenging segmentation studies. In fact, due to the flexibility of the U-Net architecture to evolve into different models, hybrid models or new U-Net architectures with different layer connections can be developed by using the block structures of other deep learning models or the proposed convolution blocks. In this context, previous studies [33,34,35,45,46] have contributed to the segmentation studies of the spinal cord and spinal cord MS lesions by developing a different perspective on the U-Net architecture. Synthesis U-Net architecture designs can be categorized as skip connection, backbone design, bottleneck, transformers, rich representation, and probabilistic design [5]. In this study, based on the innovations of backbone design and skip connection, new hybrid architectures have been developed by integrating the fractal convolutional block connection instead of the existing convolutional block structure of U-Net. In particular, the use of the logic of the fractal convolutional connection structure in the U-Net architecture and the selection of the points that affect the segmentation success of the U-Net architecture can be shown as the innovative side of the designed architectures. In addition to the FractalSpiNet architecture proposed in our previous study, in this study, the Att-FractalSpiNet architecture was developed by integrating the attention mechanism into the FractalSpiNet architecture, and the Con-FractalU-Net architecture was developed by integrating the fractal convolutional block into the skip connection in the U-Net structure.

#### 2.2.2. FractalSpiNet

FractalSpiNet is a derivative of the architecture presented in our previous work, designed by integrating fractal convolution into the backbone structure of the basic U-Net architecture for automatic detection and segmentation of spinal cord and MS lesions from MRI images [37]. The FractalSpiNet architecture goes beyond the traditional U-Net structure and increases the depth and capacity of the model by using fractal networks and multi-scale feature learning techniques. FractalSpiNet builds on the architecture of FractalNet [47] by structuring convolutional layers using a branching approach. In traditional deep learning models, network depth is increased by sequentially arranging layers, whereas in FractalSpiNet, the network structure is extended with fractal blocks containing multiple paths [37].

FractalSpiNet takes its basic structure from the U-Net architecture, as shown in Figure 3b. U-Net has an encoder-decoder structure and uses skip connections to avoid loss of detail. The difference between FractalSpiNet and U-Net is the use of fractal blocks in the encoder and decoder sections. Fractal blocks include standard convolutions, skip connections, and multi-scale attribute maps. Each block processes inputs with different path options and increases the network’s deep learning capacity. In the encoder part of FractalSpiNet, input MRI images are passed through convolutional layers, and feature maps are extracted. Multi-scale features are obtained thanks to fractal blocks. In the decoder part of the architecture, upsampling is applied to produce segmentation maps. Skip connections prevent loss of detail and preserve fine texture structures. In the final layer of FractalSpiNet, a convolutional layer with a sigmoid activation function is used to mask the spinal cord and MS lesions. Details of the basic architecture and code of the FractalSpiNet network are publicly available [48].

#### 2.2.3. Att-FractalSpiNet

In this study, in the first architecture designed for the detection and segmentation of CSC and MS lesions, the Att-FractalSpiNet model is developed by integrating the attention mechanism into the FractalSpiNet architecture, as shown in Figure 3b. The main purpose of using masks in image segmentation is to design a new layer that can determine the basic features of an image during network training and to eliminate unnecessary information by focusing on the most valuable pixel areas of the images during network training. Attention mechanisms are intermediate layer structures that determine different weights by using mask structures to determine the features of target segmentation areas in deep networks [49]. In addition, the attention mechanism allows for higher interactions and encoding of contextual information by extracting relationships between high level attributes [50]. This mechanism assigns weights to each pixel to indicate the importance of each pixel. In addition, the attention mechanism reduces computational cost by using only the relevant areas during training and provides better generalization of the network [44]. Furthermore, the attention structure can be easily integrated into standard convolutional architectures such as the U-Net model with minimal computational overhead while adding a significant boost to model sensitivity and prediction capability. With the attention connection, while the attribute information is transmitted in the U-Net model, the weak features and irrelevant regions transmitted from the data are ignored thanks to the attention block added in between, allowing the network to focus only on the area to be segmented [51].

**Figure 4:**
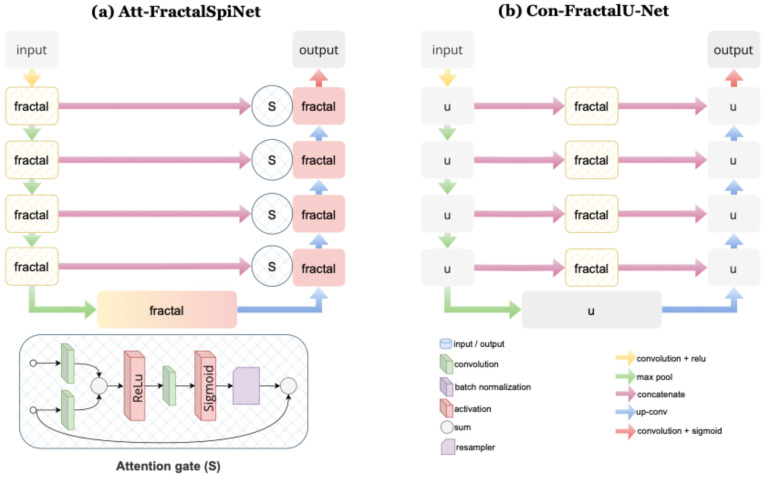
(**a**) Att-FractalSpiNet architecture developed for automatic segmentation of the CSC and cervical spinal MS lesions. This architecture is based on the FractalSpiNet architecture, which is an extended version of the U-Net architecture with a fractal convolutional network and an attention mechanism. (**b**) Detailed infrastructure of the skip connections-based Con-FractalU-Net architecture developed for segmentation of CSC and MS lesions. The Con-FractalU-Net model proposes a more efficient feature learning mechanism with fractal convolution blocks and advanced skip connections.

The detailed architecture of Att-FractalSpiNet, developed for automatic segmentation of the CSC and cervical MS lesions, is shown in Figure 4a. Att-FractalSpiNet is based on the FractalSpiNet architecture and is an extended version of the traditional U-Net architecture with a fractal convolutional network and attention mechanism. While this model is based on the encoder-decoder structure of U-Net, it enhances multiscale information learning with fractal convolutions and improves segmentation accuracy with the attention mechanism. The fractal structures allow the model to learn features at different scales without increasing the depth of the model, while the attention mechanism helps to better detect small and complex lesions. In the encoder stage of the Att-FractalSpiNet model architecture, feature extraction is performed on the input MRI image using 3 × 3 convolution, batch normalization, and ReLU activation. Fractal convolution blocks minimize the loss of detail by processing information at different scales in parallel. High level representations are produced by minimizing the spatial dimension with maximum pooling. In the decoder stage, upsampling operations restore the segmentation map to its original size. The learning capacity of the model is strengthened by skip connections, which preserve the low-level information coming from the encoder. At this stage, the attention gate is activated to ensure that the model only focuses on important regions. The attention mechanism creates a weight map by comparing the features extracted by the encoder and the decoder information. By filtering out noisy or redundant information, the model improves segmentation accuracy and can determine more precise boundaries.

#### 2.2.4. Con-FractalU-Net

In Con-FractalU-Net, the second architecture developed for cervical spine and MS lesion detection and segmentation, a fractal convolution block is integrated into the skip connection in the U-Net structure, as shown in Figure 3a. Skip connections were first proposed in U-Net to address the problem of model performance degradation with increasing depth of the architecture [52]. Skip connections can be expressed as using connections from certain convolution outputs of the model as inputs to different points of the model instead of sequential connections. Skip connections are used to transfer the convolution outputs to the opposite layers before the pooling process, while the downward convolution process takes place. In this connection, the location information of the features coming from the encoder section and the high feature information in the decoder section are combined to produce an output. In contrast to the successive connection structure used in deep networks, skip connections have been used in image segmentation studies [53,54,55]. It is known that the architecture with skip connections has a better generalization ability than the architecture without skip connections [56]. In fact, the main structure that distinguishes the U-Net architecture from other sequential processing deep networks is that it has skip connections. It has been observed that U-Net-based hybrid architectures with skip connections achieve similar results to the basic U-Net architecture in pixel-based segmentation tasks [57,58,59,60,61].

Figure 4b shows the detailed infrastructure of the Con-FractalU-Net architecture developed for segmentation of CSC and MS lesions. Based on the traditional U-Net structure, this model provides a more efficient feature learning mechanism with fractal convolution blocks and advanced skip connections. Fractal convolution blocks increase the capacity of multi-scale information processing to produce more detailed feature maps, while extended skip connections minimize the loss of detail by improving the flow of information between the encoder and decoder. This enables more accurate segmentation, particularly of small and complex MS lesions. In the encoder stage of the Con-FractalU-Net architecture, the input MRI image is passed through several convolutional layers to extract detailed features. At each level, deep and multi-scale feature learning is performed using fractal structures. The maximum pooling process reduces the image size to create more abstract and meaningful representations. This process allows better recognition of the complex tissue and lesion structures of the model. In the decoder stage, the image size is increased again by upsampling, and segmentation output is generated. The main innovation of Con-FractalU-Net is the use of extended and optimized skip connections, in contrast to conventional U-Net.

## 3. Results

In this study, experimental studies were carried out with two new proposed architectures, Con-FractalU-Net and Att-FractalSpiNet, for automatic segmentation of the cross-sectional area (CSA) of the CSC area and detection of MS lesions in the CSC. Furthermore, the results obtained with these architectures are compared with the results of the basic U-Net architecture and our proposed FractalSpiNet architecture. The experimental studies are also carried out using the T2-w MRI dataset [39], which was created for our previous work [37] and is publicly available. The experimental studies for automatic segmentation of the CSC and detection of MS lesions in the CSC were performed using the workstation computer whose specifications are given in Table 1. The Con-FractalU-Net and Att-FractalSpiNet deep learning architectures proposed in this study and the implementations of the basic U-Net and FractalSpiNet architectures were carried out in the Jupyter Notebook IDE environment (v. 7.1.2) on TensorFlow (v. 2.6.0) using the Python programming language (v. 3.6.13).

The dataset used in this study contains a total of 231 axial MR slices obtained from MS patients suitable for experimental studies. As in our previous study [37], the same image pre-processing procedures were applied to the MR images in the database. Although the number of images in the dataset is small, the CSA region and MS lesions in the MRI slices are quite unique in terms of location and shape, which can be considered a positive situation in the data augmentation process. For better learning of the proposed networks and to avoid the overfitting effect, this number was increased to a total of 1080 using data augmentation techniques. As data augmentation techniques, the image set was augmented by using rotation (on x and y axes), flipping, shifting, and same functions, which are based on geometric transformation without disturbing the pixel structure. Data augmentation techniques were applied to both the MRI slices and the ground truth masks in the dataset. In addition, data augmentation was performed in the Python environment using the NumPy library (v. 1.19.5).

**Figure 5:**
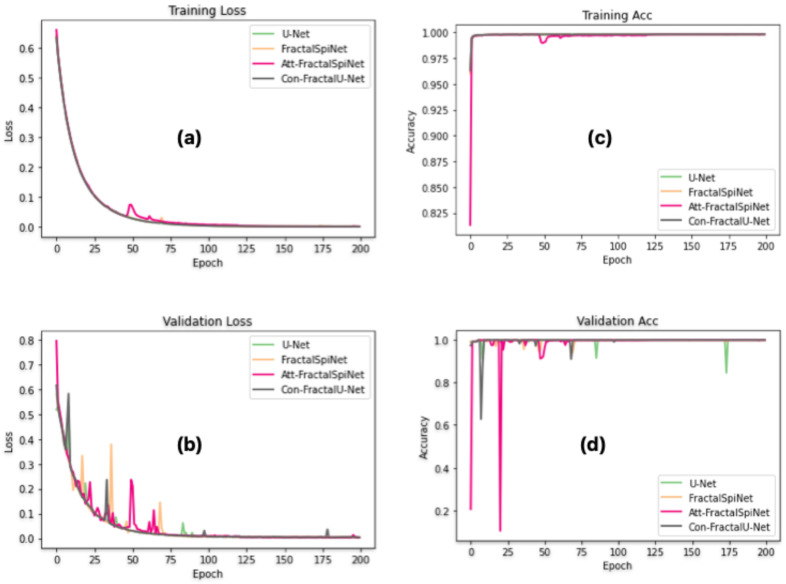
Training and validation performance of U-Net, FractalSpiNet, Att-FractalSpiNet, and Con-FractalU-Net for CSC and MS lesion segmentation for 200 epochs. (**a**) training loss, (**b**) validation loss, (**c**) training accuracy, and (**d**) validation accuracy.

After the data augmentation process, 80% (864) of the total 1080 MRI images in the dataset were used for training the U-Net, FractalSpiNet, Att-FractalSpiNet, and Con-FractalU-Net architectures, and the remaining 20% (216) were used for testing. Some of the images in the training set were used for validation. The progression of training loss, validation loss, training accuracy (Training Acc), and validation accuracy (Validation Acc) values obtained as a result of training the proposed U-Net, FractalSpiNet, Att-FractalSpiNet, and Con-FractalU-Net architectures for CSC segmentation and detection of MS lesions along the spinal cord using axial MRI images in the dataset over 200 epochs are shown in Figure 5a, Figure 5b, Figure 5c, and Figure 5d, respectively. The plots of these values provide important information about the performance, generalizability, and potential problems of the model.

In Figure 5a, the training loss of all models decreases steadily as the number of epochs increases, indicating effective learning. The initial rapid decline in loss suggests that the models adapt quickly to the dataset, while the later stabilization implies convergence. Among the models, FractalSpiNet and Att-FractalSpiNet show slightly higher initial losses compared to U-Net and Con-FractalU-Net, likely due to their increased architectural complexity. Figure 5b shows the validation loss, which exhibits more fluctuations compared to training loss. The fractal-based architectures generally demonstrate lower validation loss over time, suggesting improved generalization compared to the baseline U-Net. For the training accuracy in Figure 5c, all architectures reach high accuracy values that converge close to 1.0, which indicates that they fit well to the training data. The overall consistency between the models suggests that all architectures effectively learn the segmentation task on the training dataset. In Figure 5d, the validation accuracy is plotted. While U-Net exhibits some fluctuations, FractalSpiNet, Con-FractalU-Net, and Att-FractalSpiNet achieve higher and more stable validation accuracy throughout the training process. Overall, the results suggest that while U-Net provides a stable baseline, the proposed fractal-based architectures enhance the segmentation capabilities by improving generalization performance. These findings confirm the effectiveness of incorporating fractal structures and attention mechanisms in deep learning-based segmentation of CSC and MS lesions.

This study also compares different segmentation architectures in terms of training time and model complexity. The baseline U-Net model, with 31.4 million parameters, demonstrated the shortest training time of 28 min and 37 s. FractalSpiNet, which incorporates fractal-based structures, significantly increased the number of parameters to approximately 109.9 million, resulting in a longer training time of 91 min and 18 s. Among the proposed hybrid architectures, Att-FractalSpiNet, which integrates an attention mechanism with fractal structures, had the highest parameter count (115.8 million) and the longest training time (99 min and 52 s), reflecting the computational cost of the attention modules. In contrast, Con-FractalU-Net, designed with enhanced skip connections within a fractal framework, maintained a more balanced trade-off with 53.3 million parameters and a training time of 60 min and 5 s. These results highlight the impact of architectural modifications on computational efficiency, showing that while attention-based enhancements contribute to improved segmentation, they require higher computational resources.

To ensure a fair and consistent evaluation of the U-Net, FractalSpiNet, Con-FractalU-Net, and Att-FractalSpiNet architectures, all models were trained using the same set of hyperparameters. These hyperparameters were carefully chosen to ensure robust training while maintaining computational efficiency, making them suitable for evaluating the segmentation performance of each proposed architecture under standardized conditions. The training process was performed over 200 epochs to allow sufficient learning and convergence while mitigating the risk of underfitting. A batch size of 8 was chosen to balance memory efficiency and gradient update stability. The learning rate was set to 0.001, a value commonly used in deep learning segmentation tasks, to ensure steady convergence without drastic fluctuations in weight updates. A dropout rate of 0.5 was employed to prevent overfitting by randomly deactivating neurons during training, thereby enhancing the model’s generalization ability. For activation functions, ReLU was chosen as the primary nonlinearity because it efficiently mitigates the vanishing gradient problem, allowing deeper networks to learn effectively. At the output layer, the sigmoid activation function was used, as the segmentation task is formulated as a binary classification problem at the pixel level. The Adam optimization algorithm was utilized due to its adaptive learning rate properties, which facilitate faster and more stable convergence compared to traditional methods such as stochastic gradient descent (SGD). The binary cross-entropy loss function was employed, aligning with the binary nature of the segmentation task, ensuring proper gradient updates for foreground and background pixel classification.

In the experimental studies, the metrics in a previous study [37] were used to evaluate the performance of CSC segmentation and the detection of MS lesions. The evaluation was based on pixel overlap, volume difference, and geometric distance measurements. To measure segmentation accuracy, the Dice Similarity Coefficient (DSC) in Equation (1) was used. DSC evaluates the spatial overlap between the predicted mask (PM) and the ground truth mask (GT), where higher values indicate better segmentation performance. For volume-based evaluation, the Volume Overlap Error (VOE) in Equation (2) and Relative Volume Difference (RVD) in Equation (3) metrics were employed. VOE quantifies the proportion of segmentation errors, while RVD represents the percentage difference between the predicted and actual segmentation volumes [62,63]. To assess geometric accuracy, the Average Surface Distance (ASD) in Equation (4), Hausdorff Distance (HD) in Equations (5) and (6), and Hausdorff 95 (HD95) in Equation (7) were utilized. ASD measures the accuracy of segmentation boundaries, while HD calculates the maximum point-wise distance between one segmentation and another. HD95 refines this measurement by considering the 95th percentile, reducing the impact of outliers. For lesion detection performance, recall (REC) in Equation (8) and precision (PRE) in Equation (9) were used. REC measures the proportion of actual lesion pixels correctly identified, whereas PRE evaluates the correctly detected lesions while minimizing false positives.

(1) $$DSC(PM,GT)=\frac{2\left|PM\cap GT\right|}{\left|PM\right|\cup \left|GT\right|}\times 100$$

(2) $$VOE\left(PM,GT\right)=(1-\frac{\left|PM\cap GT\right|}{\left|PM\right|+\left|GT\right|-\left|PM\cup GT\right|})\times 100$$

(3) $$RVD\left(PM,GT\right)=(\frac{\left|PM\right|-\left|GT\right|}{\left|GT\right|})\times 100$$

(4) $$ASD\left(PM,GT\right)=\frac{1}{\left|s\left(PM\right)\right|+\left|s\left(GT\right)\right|}\left(\begin{array}{c} \sum_{S_{PM}\in S(PM)}d\left(SPM,S\left(GT\right)\right)+\\ \sum_{S_{GM}\in S(GT)}d\left(SGM,S\left(PM\right)\right) \end{array}\right)$$

(5) $$hd\left(PM,GT\right)=\underset{x\in \mathrm{PM}}{\max}\underset{y\in \mathrm{GM}}{\min}{\left\|x-y\right\|}_{2}$$

(6) $$hd(GT,PM)=\underset{y\in \mathrm{GM}}{\max}\underset{x\in \mathrm{PM}}{\min}{\left\|x-y\right\|}_{2}$$

(7) $$HD95\left(PM,GT\right)=\max\left(hd(PM,GT),hd(GT,PM\right))$$

(8) $$REC(PM,GT)=\frac{TP}{TP+FN}\times 100$$

(9) $$PRE\left(PM,GT\right)=\frac{TP}{TP+FP}\times 100$$

In this study, four deep learning architectures—U-Net, FractalSpiNet, Att-FractalSpiNet, and Con-FractalU-Net—were trained under identical conditions using axial-plane CSC MRI images to segment two key regions: the cross-sectional area (CSA) of the spinal cord and MS lesions within this area. The models were trained for 200 epochs, and their performance was evaluated using several segmentation metrics, as shown in Table 2. Among these architectures, Con-FractalU-Net achieved the highest DSC of 98.89%, VOE of 2.05%, and the lowest ASD of 1.09 mm, making it the most accurate model for segmentation. Additionally, its PRE of 99.21% indicates a strong ability to minimize false positives. FractalSpiNet also demonstrated strong performance with a DSC of 98.88%, the lowest VOE (2.04%), and ASD (1.38 mm), while maintaining a high REC of 98.84%, suggesting robust lesion detection capability. On the other hand, Att-FractalSpiNet, with a DSC of 98.41%, exhibited higher segmentation boundary errors, as indicated by its ASD (2.73 mm) and HD95 of 0.80 mm, suggesting that the attention mechanism introduced more variability. The baseline U-Net, while achieving a DSC of 98.54%, lagged behind in all key metrics, reaffirming the superiority of fractal-based architectures. Overall, Con-FractalU-Net emerges as the most effective model, demonstrating superior segmentation accuracy and precision, while FractalSpiNet remains a strong alternative with competitive performance. These findings highlight the advantages of fractal-based networks in enhancing segmentation robustness and accuracy for the CSC MRI dataset.

**Figure 6:**
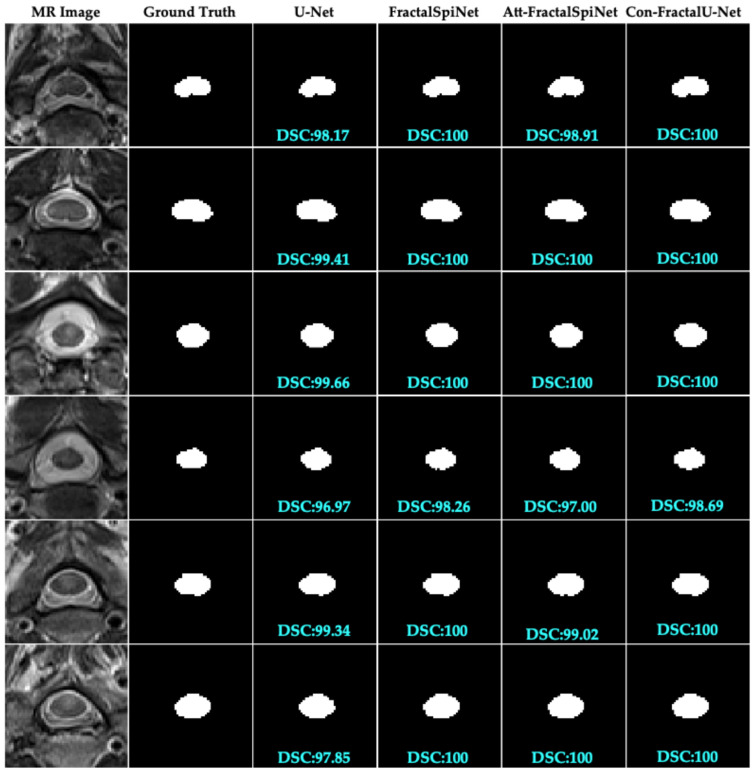
Comparison of CSC cross-sectional segmentation results obtained using U-Net, FractalSpiNet, Att-FractalSpiNet, and Con-FractalU-Net models.

In this study, we compare the performance of the deep learning architectures U-Net, FractalSpiNet, Att-FractalSpiNet, and Con-FractalU-Net for segmentation of CSC MR images. Figure 6 shows the segmentation results for only a part of the test dataset but provides important information about the general trends of the different architectures. In the figures, the segmentation success of each model is evaluated using DSC scores. Con-FractalU-Net demonstrated excellent segmentation performance, reaching 100% for DSC in all test images shown. This shows that the model is able to recognize both spinal cord cross-sectional area (CSA) and MS lesions with very high accuracy and successfully generalize the features learned during training. Similarly, the FractalSpiNet model also achieved a score of 100% within DSC in most cases but was slightly below this value in some images. Although Att-FractalSpiNet includes an attention mechanism to improve segmentation performance, DSC ≈ 98–99% in some test images. This result suggests that while the attention mechanism may be advantageous in certain situations, it may not be sufficient to achieve perfect segmentation in some cases. U-Net was the model with the lowest performance compared to the other architectures. In some images, the DSC value fell below 98% and showed lower accuracy compared to other models, especially in complex boundary regions. This indicates the limitations of the typical U-Net architecture for CSC segmentation, and fractal-based models, which are more advanced structures, appear to be more successful. Together with the full analysis of all test images, Con-FractalU-Net provides the most stable and highly accurate segmentation model. It has been observed that fractal-based networks are more successful than classical CNN-based models and increase segmentation accuracy, especially in medical imaging applications where high precision is required.

Segmentation of MS lesions is a more challenging task than determining the cross-sectional area of the spinal cord, and accurate detection of lesions is a significant challenge due to their small volume and variable morphology. While the cross-sectional area of the spinal cord is already a very small pixel area, MS lesions have an even smaller and more challenging volumetric structure within this small area. Table 3 shows the segmentation performance of the U-Net, FractalSpiNet, Att-FractalSpiNet, and Con-FractalU-Net models on MS lesions. In terms of the DSC metric, the Con-FractalU-Net model has the highest success rate with 91.48%, followed by FractalSpiNet with 90.90%. In particular, the Con-FractalU-Net model provided the most accurate segmentation with low ASD and HD95 values, allowing better delineation of MS lesion boundaries. On the other hand, the U-Net model showed the lowest segmentation performance with a DSC value of 86.00% and high values, especially for the ASD and HD95 metrics, indicating that the model was less successful in identifying lesion boundaries compared to other models. While the Att-FractalSpiNet model performed competitively with a DSC value of 88.79%, it has a higher value in the ASD metric compared to the other models, indicating that segmentation performance may be lower in some cases. The Con-FractalU-Net model showed the best performance in terms of the REC metric, while this model also showed the best performance in terms of PRE. In conclusion, the Con-FractalU-Net model stands out as the most successful method for segmentation of MS lesions in terms of general metrics. The FractalSpiNet model also shows competitive performance with high DSC and low error rates. U-Net, on the other hand, performed poorly compared to the other models in terms of segmentation accuracy. These results suggest that Con-FractalU-Net is a more reliable model for MS lesion segmentation.

**Figure 7:**
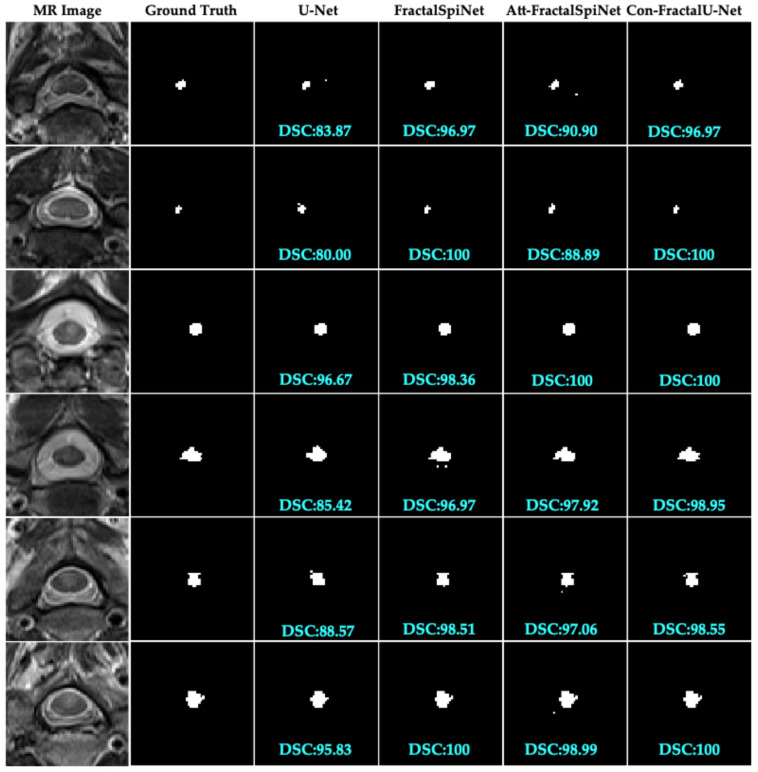
Performance comparison and DSC scores of U-Net, FractalSpiNet, Att-FractalSpiNet, and Con-FractalU-Net architectures in segmentation of MS lesions in CSC.

In this study, the performance of different deep learning architectures for the automatic segmentation of MS lesions in the CSC was compared, as shown in Figure 7. The segmentation results obtained using U-Net, FractalSpiNet, FractalSpiNet with an attention mechanism (Att-FractalSpiNet), and FractalU-Net with convolutional blocks (Con-FractalU-Net) architectures were evaluated using the DSC metric. The results revealed that the typical U-Net architecture exhibited relatively lower performance in segmenting MS lesions, while FractalSpiNet and especially Att-FractalSpiNet and Con-FractalU-Net architectures had a significantly better performance. By producing consistent and accurate segmentation results with high DSC values, fractal-based architectures emerge as more promising approaches for automated analysis of MS lesions. Notably, the integration of attention mechanisms and convolutional blocks significantly improved the model’s ability to segment and detect lesions, achieving DSC values exceeding 98% and even reaching 100%. The findings of this study strongly support that advanced architectures, in particular Att-FractalSpiNet and Con-FractalU-Net, can deliver significant performance gains in medical imaging, especially for complex and detail-demanding tasks. The significant improvement achieved compared to the standard U-Net architecture reveals the potential of these advanced architectures to automatically and accurately segment challenging structures such as MS lesions in the CSC.

## 4. Discussion

This study presents a comparative analysis of advanced segmentation architectures for the detection of MS lesions in the CSC and segmentation of the CSA region. Our previous work introduced FractalSpiNet [37] as an effective architecture, demonstrating high segmentation performance. Building on this foundation, we propose two novel architectures, Con-FractalU-Net and Att-FractalSpiNet, which aim to further improve segmentation performance by incorporating enhanced skip connections and attention mechanisms, respectively. Experimental results confirm that Con-FractalU-Net achieves the highest segmentation accuracy across both tasks. Specifically, for MS lesion segmentation, Con-FractalU-Net achieved the best DSC score (91.48%), outperforming FractalSpiNet (90.90%), Att-FractalSpiNet (88.79%), and the baseline U-Net (86.00%). The improved connectivity introduced in Con-FractalU-Net is likely to contribute to its superior performance, ensuring better feature propagation and refinement. Similarly, in CSA segmentation, Con-FractalU-Net showed improved accuracy over all other architectures, reinforcing its robustness across different segmentation tasks.

A key aspect of this study is the comparison of training efficiency and computational complexity between different architectures. The baseline U-Net, with 31.4 million parameters, had the shortest training time (28 min and 37 s). FractalSpiNet, which introduced fractal-based structures, significantly increased the number of parameters to approximately 109.9 million, leading to an extended training time of 91 min and 18 s. Att-FractalSpiNet, which integrates attention mechanisms into the fractal framework, had the highest number of parameters (115.8 million) and the longest training time (99 min and 52 s), reflecting the additional computational cost of the attention modules. In contrast, Con-FractalU-Net maintained a more balanced trade-off between accuracy and computational efficiency, with 53.3 million parameters and a training time of 60 min and 5 s. This demonstrates that while attention-based enhancements improve segmentation quality, they require significantly more computational resources, making Con-FractalU-Net a preferable option in scenarios where both accuracy and efficiency are crucial. Analyses of time performance in the test set reveal that deep learning architectures generally exhibit high efficiency in the process of detecting MS lesions in CSC. When evaluating the entire test set of 216 MR images, the overall detection times for all methods were obtained to be within a close range. FractalSpiNet emerged as the fastest method with a minimally different total test set processing time of 44.42 s and an average detection time of 0.205 s per image. U-Net was recorded as the slowest, with a total time of 45.51 s and an average time of 0.211 s. The Con-FractalU-Net and Att-FractalSpiNet architectures, on the other hand, showed similar performance in the mid-range with total times of 44.99 s and 44.92 s, respectively, and an average time of 0.208 s for both. The fact that the average detection times for a single image are less than a quarter of a second for all methods and that the total processing times for the test set are around 45 s demonstrates that all architectures are sufficiently time-efficient for practical clinical applications. When these results are evaluated together with previous accuracy analyses, they support that the developed fractal-based architectures have a strong potential for clinical use by offering both high accuracy and efficient processing times in the automatic segmentation of MS lesions.

The results also indicate that MS lesion segmentation is inherently more challenging than CSA segmentation due to the smaller size and irregular distribution of lesions. Despite this complexity, the proposed architectures, particularly Con-FractalU-Net, successfully improved segmentation performance compared to the baseline U-Net and previously developed FractalSpiNet [37]. The ability to effectively segment both MS lesions and CSA highlights the adaptability and robustness of the proposed fractal-based architectures. For segmentation of cervical spinal cord and spinal cord MS lesions, the Con-FractalU-Net architecture proposed in this study is slightly more successful than the Att-FractalSpiNet architecture in terms of the DSC metric. To evaluate the effect of the Att-FractalSpiNet architecture proposed in this study and the underlying attentional mechanism, it is necessary to review the results of our previous study [37]. For CSA segmentation in CSC, on the same dataset in our previous study [37], 98.01% and 97.90% DSC scores were achieved using the Att U-Net and Att-Res U-Net architectures based on the attention mechanism and residual, respectively, while 98.41% DSC scores were achieved using the Att-FractalSpiNet architecture in this study. On the other hand, although 75.34% and 83.06% DSC scores were achieved using Att U-Net and Att-Res U-Net architectures to detect MS lesions in the cervical spinal cord, respectively, the detection of MS lesions using the Att-FractalSpiNet architecture proposed in this study was achieved with a DSC score of 88.79%. Thus, the fact that the attention mechanism integrated into the fractal structure in the proposed architectures achieves higher scores than the residual structure shows the effect and effectiveness of the attention mechanism on the architectures.

The proposed Con-FractalU-Net and Att-FractalSpiNet models in this study demonstrate significant improvements in CSA segmentation and MS lesion detection compared to state-of-the-art methods in previous studies. For spinal cord segmentation, the PropSeg method introduced by De Leener et al. [19] achieved a DSC of 91.0% for spinal cord and spinal canal segmentation, while the U-Net-based segmentation by Bedard et al. [64] improved the DSC score to 96.0%. In addition, McCoy et al. [23] obtained 93.0% DSC for segmentation of the spinal cord using 2D CNN architecture. For CSA segmentation, the OPAL algorithm and STEPS segmentation process by Prados et al. [65] achieved 96.5% DSC for CSA segmentation with visible lesions and 97.0% DSC without visible lesions. The U-Net-based model from Zhang et al. [26] achieved 87.0% DSC, while the channel-attentive U-Net (SeUneter) by Zhang et al. [35] reached 90.67% DSC. In comparison, the proposed Att-FractalSpiNet (98.41% DSC) and Con-FractalU-Net (98.89% DSC) further enhanced segmentation results, outperforming previous approaches. For MS lesion detection, different models in the literature show varying performance. The CNN (DeepSeg) model from Gros et al. [22] obtained 60.4% DSC, while the MultiResUNet model by Zhuo et al. [45] achieved 50.0% DSC for MS lesion segmentation. The residual attention-aware U-Net from Bueno et al. [36] demonstrated 90.4 ± 0.101% DSC for CSC segmentation. On the other hand, Karthik et al. [30] achieved a DSC score of 72.0% in automated segmentation of MS lesions in the spinal cord. The proposed models further improved these results, with Att-FractalSpiNet reaching 88.79% DSC and Con-FractalU-Net achieving 91.48% DSC, making them the most effective solutions for MS lesion segmentation. Compared to other advanced approaches, the FractalSpiNet model by Polattimur et al. [37], our previous study, achieved 98.88% DSC for CSA segmentation and 90.90% DSC for MS lesion detection. While this model demonstrated strong performance, the proposed Con-FractalU-Net (98.89% DSC for CSA, 91.48% DSC for MS) further enhanced segmentation accuracy, establishing them among the best-performing models in the previous. Additionally, the 2D and 3D CNN-based model by Naga Karthik et al. [30] achieved 72.0% DSC for MS lesion detection, which was significantly outperformed by the proposed models. Overall, the proposed Con-FractalU-Net and Att-FractalSpiNet models achieve the highest accuracy in CSA segmentation and MS lesion detection, positioning them as state-of-the-art methods. These findings demonstrate that integrating fractal-based architectures, attention mechanisms, and skip connections leads to substantial improvements in CSC segmentation and MS lesion detection, surpassing existing approaches. Statistical significance tests were also performed using the Wilcoxon signed-rank test to compare the results of Con-FractalU-Net, which obtained the highest DSC score for automatic segmentation of the cervical spinal cord, with FractalSpiNet, Att-FractalSpiNet, and the other studies mentioned above. In the automatic segmentation of the spinal cord, a *p*-value of 0.0312 was obtained in the Wilcoxon signed-rank test comparing Con-FractalU-Net with other methods in terms of statistical significance. Similarly, the *p*-value of 0.0039 was observed in the comparison of the proposed Con-FractalU-Net, which is the most successful method compared to the other methods in terms of statistical significance in the detection and segmentation of MS lesions in the cervical spinal cord. Since the *p*-value is less than 0.05 in both evaluations, it is confirmed that the results achieved with Con-FractalU-Net are statistically significant.

## 5. Conclusions

Early and accurate detection of MS lesions in the CSC is critical for patient care. Manual segmentation is a challenging and error-prone process, even for experts, as lesions can be very small and subtle in spinal cord MR images. In this context, deep learning architectures offer a promising alternative by potentially increasing diagnostic accuracy and efficiency for radiologists, reducing manual segmentation time, and enhancing diagnostic confidence. The segmentation of MS lesions in the CSC presents unique challenges due to the complex anatomical structure of the spinal cord, variations in lesion morphology, and limitations in MR imaging quality. The spinal cord does not possess a uniform geometric shape, and its boundaries change dynamically along its length, making accurate segmentation particularly difficult. In addition, MS lesions exhibit significant heterogeneity in size, shape, and location, which adds to the diversity of the dataset but also adds complexity to the segmentation process.

In this study, we proposed two novel deep learning architectures, Con-FractalU-Net and Att-FractalSpiNet, to improve the segmentation of CSA and MS lesions in the CSC by leveraging fractal-based structures, skip connections, and attention mechanisms. These architectures were compared against the previously introduced FractalSpiNet and the baseline U-Net model. The results demonstrate that incorporating fractal elements improves segmentation performance by allowing multi-scale feature extraction, while the addition of attention modules further refines spatial awareness in lesion localization. Our findings indicate that Con-FractalU-Net achieved the highest overall performance across all evaluation metrics, with a DSC of 91.48%, outperforming the other architectures. Att-FractalSpiNet, although slightly lower in DSC (88.79%), showed robust precision and recall values, indicating its effectiveness in lesion identification. Beyond lesion segmentation, CSA segmentation was also evaluated, as it plays a crucial role in contextualizing MS lesion burden and progression. The results showed that Con-FractalU-Net and FractalSpiNet effectively segmented the CSA with high DSC values, demonstrating their ability to generalize well to spinal cord structures. This is particularly important for clinical applications, where accurate delineation of both CSA and lesions helps to monitor disease progression and treatment response.

The computational efficiency of the models was also evaluated. While the baseline U-Net had the shortest training time, the proposed architectures required additional computational resources. Con-FractalU-Net provided a more balanced trade-off, making it a computationally efficient alternative without compromising segmentation accuracy. From a clinical perspective, the improved accuracy of MS lesion segmentation in the CSC has significant implications. Precise segmentation allows for more reliable lesion volume quantification, which is crucial for assessing disease activity and treatment efficacy. Improved segmentation models, such as Con-FractalU-Net, can be integrated into radiological workflows to support automated MS lesion detection, reducing the variability associated with manual annotations and improving diagnostic consistency across clinicians.

Although the proposed architectures have shown promising results, several areas warrant further investigation to improve their applicability and robustness. While the fractal-based architectures improve segmentation accuracy, their computational cost remains a limiting factor. One of the critical challenges in this study is the relatively small size of the dataset, which may limit the generalizability and robustness of the proposed models. Although high segmentation performance was achieved with the fractal-based architectures, models trained on small datasets are prone to overfitting and may not perform consistently on unseen data from different institutions or imaging protocols. To address this limitation, future work will focus on the integration of transfer learning techniques, where pre-trained weights on large medical image datasets could be used to improve learning efficiency and generalization. In addition, cross-institutional validation using external datasets from other medical centers is planned to further assess the robustness and adaptability of the proposed models. These steps are essential to ensure clinical applicability in real-world settings and to confirm that segmentation performance is maintained across different imaging conditions and patient populations. Future work may also explore model pruning, quantization, and knowledge distillation techniques to reduce model complexity while maintaining performance. In addition, expanding the evaluation to larger and more diverse datasets, including multi-center clinical MRI scans, can help validate the models’ robustness across different imaging protocols and scanner variations. The ultimate goal is to translate these models into clinical practice. In addition, future work may include prospective studies where automated segmentation results are validated against expert radiologists’ annotations in a real-world clinical setting.

## Figures and Tables

**Table 1:** Workstation computer and its specifications used in experimental studies for automatic segmentation of the CSC and detection of MS lesions in the CSC.

Hardware/Component	Specification
Computer	Workstation
Mainboard	Gigabyte B560M
Central processor (CPU)	Intel Core i5 4.10 GHz
Memory (RAM)	16 GB DDR4 3000 MHz
Graphical processing unit (GPU)	NVIDIA RTX A4000 16 GB
Disk drivers	1TB HDD + 500 GB SSD

**Table 2:** Performance comparison of deep learning architectures such as U-Net, FractalSpiNet, Att-FractalSpiNet, and Con-FractalU-Net for CSA segmentation of CSC using MRI scans.

Architectures	DSC (%)	VOE (%)	RVD (%)	ASD [mm]	HD95 [mm]	REC (%)	PRE (%)
U-Net	98.54	2.67	1.51	1.67	0.49	98.43	98.69
FractalSpiNet	98.88	2.04	0.97	1.38	0.39	98.84	98.94
Att-FractalSpiNet	98.41	2.84	1.57	2.73	0.80	98.75	98.11
Con-FractalUNet	98.89	2.05	1.18	1.09	0.51	98.62	99.21

**Table 3:** Performance comparison of U-Net, FractalSpiNet, Att-FractalSpiNet, and Con-FractalU-Net architectures for MS lesion segmentation in CSC using MRI scans.

Architectures	DSC (%)	VOE (%)	RVD (%)	ASD [mm]	HD95 [mm]	REC (%)	PRE (%)
U-Net	86.00	20.83	13.50	28.23	11.55	83.73	90.50
FractalSpiNet	90.90	14.06	9.62	16.08	8.06	91.26	92.20
Att-FractalSpiNet	88.79	17.17	11.77	35.81	11.59	89.33	89.79
Con-FractalU-Net	91.48	12.92	9.93	20.84	7.27	92.13	92.27

## Data Availability

The raw data supporting the conclusions of this article is publicly available from Mendeley Data (https://data.mendeley.com/datasets/ydkrtmygjp/1, accessed on 9 March 2025).
